# A UML profile for the OBO relation ontology

**DOI:** 10.1186/1471-2164-13-S5-S3

**Published:** 2012-10-19

**Authors:** Gabriela DA Guardia, Ricardo ZN Vêncio, Cléver RG de Farias

**Affiliations:** 1 Department of Computer Science and Mathematics (DCM/FFCLRP), University of São Paulo (USP) Av. Bandeirantes, 3900 - Monte Alegre - Ribeirão Preto - SP - 14040-901 - Brazil

## Abstract

**Background:**

Ontologies have increasingly been used in the biomedical domain, which has prompted the emergence of different initiatives to facilitate their development and integration. The Open Biological and Biomedical Ontologies (OBO) Foundry consortium provides a repository of life-science ontologies, which are developed according to a set of shared principles. This consortium has developed an ontology called OBO Relation Ontology aiming at standardizing the different types of biological entity classes and associated relationships. Since ontologies are primarily intended to be used by humans, the use of graphical notations for ontology development facilitates the capture, comprehension and communication of knowledge between its users. However, OBO Foundry ontologies are captured and represented basically using text-based notations. The Unified Modeling Language (UML) provides a standard and widely-used graphical notation for modeling computer systems. UML provides a well-defined set of modeling elements, which can be extended using a built-in extension mechanism named Profile. Thus, this work aims at developing a UML profile for the OBO Relation Ontology to provide a domain-specific set of modeling elements that can be used to create standard UML-based ontologies in the biomedical domain.

**Results:**

We have studied the OBO Relation Ontology, the UML metamodel and the UML profiling mechanism. Based on these studies, we have proposed an extension to the UML metamodel in conformance with the OBO Relation Ontology and we have defined a profile that implements the extended metamodel. Finally, we have applied the proposed UML profile in the development of a number of fragments from different ontologies. Particularly, we have considered the Gene Ontology (GO), the PRotein Ontology (PRO) and the Xenopus Anatomy and Development Ontology (XAO).

**Conclusions:**

The use of an established and well-known graphical language in the development of biomedical ontologies provides a more intuitive form of capturing and representing knowledge than using only text-based notations. The use of the profile requires the domain expert to reason about the underlying semantics of the concepts and relationships being modeled, which helps preventing the introduction of inconsistencies in an ontology under development and facilitates the identification and correction of errors in an already defined ontology.

## Background

Researches in many fields of knowledge have benefited from the use of representational resources such as terminologies, controlled vocabularies and ontologies. These resources are used not only to facilitate the development of computer systems but also to assist in the automated execution of different tasks by these systems. In the biomedical area the use of these resources has gained more visibility due to the rapidly growing volume of information. Ontologies are especially able to support the biomedical research in many different ways [1,2]. The main applications of ontologies in the biomedical domain include the provision of a (standard) vocabulary for the functional annotation of biological data [3-6], the support for information retrieval across databases and biomedical literature [7-10], and standardization and integration of information and computer systems in the domain [11-14].

Despite the increasingly use of ontologies in the biomedical domain, there are a number of challenges that hinder their effectiveness. These challenges include the use of different languages and approaches for ontology representation [15,16] and the development of methods for ontology quality evaluation [2,17-19]. The need to integrate different ontologies, often pertaining to the same domain, poses an additional challenge [20-23]. As a consequence, a number of initiatives towards ontology development standardization have emerged.

The Open Biological and Biomedical Ontologies (OBO) Foundry is a consortium that provides a repository of life-science ontologies [24]. These ontologies are developed according to a set of shared principles, including openness, orthogonality, collaborative development and the use of a well-defined syntax and common relations. The OBO Foundry ontologies include the Gene Ontology (GO) [3], the Chemical Entities of Biological Interest Ontology (ChEBI) [25], the Phenotypic Quality Ontology (PATO) [26], the PRotein Ontology (PRO) [27] and the Xenopus Anatomy and Development Ontology (XAO) [28], among others. Additionally, the Foundry also includes a number of candidate ontologies and other ontologies of interest in the life-science domain.

Ontologies curated by the OBO Foundry are basically represented using text-based notations, viz., the OBO Flat File Format [29] and the Web Ontology Language (OWL) [30,31]. OWL is an ontology definition language originally conceived for the semantic web. OWL specifications are serialized using a machine-readable RDF/XML-based format [32], i.e., owl specifications are exchanged as RDF documents. The OBO Flat File Format or simply OBO format is also a machine-readable, text-based ontology representation language. The OBO format provides a subset of the concepts in OWL, with a number of extensions. OBO Foundry ontologies are usually developed and maintained as OBO format and automatically converted into OWL.

Another OBO Foundry initiative to foster ontology development and integration was the development of the OBO Relation Ontology [33,34]. The OBO Relation Ontology consists of a rigorously-defined set of relations commonly used in biomedical ontologies. The development of such ontology aims at providing consistent and unambiguous formal definitions for relations used to connect biological entity classes in biomedical ontologies. A class describes common characteristics to a set of biological entities existing in the real world. The use of relations and classes in a manner consistent with the definitions provided by the OBO Relation Ontology facilitates the analysis and integration of biomedical knowledge represented in different ontologies using computational tools.

Some authors argue that conceptual modeling artifacts, such as ontologies, are (primarily) intended to be used by humans, not machines [35-37]. Mi and Thomas also argue that in order to succeed, ontologies and standards in bioscience should be designed not only to be readable by computers, but also to be accurate and intuitive to (human) biologists [38]. Visual (graphical) languages and formalisms have long been used to represent knowledge in complex (biological) systems. Graphical models are generated, communicated and comprehended by humans. Still, these models can be manipulated, maintained and analysed by computer systems [39]. Thus, efforts have been made to develop graphical notations (diagrams) to represent biological knowledge [40-43].

The Unified Modeling Language (UML) [44,45] is a standard graphical language widely used in the specification, documentation and visualization of computer artifacts and ontologies. UML wide acceptance is due to its semantically rich and well-defined set of modeling concepts, its independence of specific methodologies and a wide range of supporting tools. Additionally, UML has a built-in extension mechanism named Profile that can be used to provide new modeling elements specific to a particular domain, which facilitates its adoption and use across different application domains.

Despite the broad acceptance of UML by the software engineering and conceptual modeling communities, little support is provided by the OBO Foundry for the development of ontologies using graphical notations in general and using UML in particular. OBO Foundry ontologies can be developed using the OBO-Edit [46], an open source ontology editor that supports the OBO format. However, no support is provided for UML.

This work aims at developing a UML profile for the OBO Relation Ontology. We have studied the different types of biological entity classes and relations defined in this ontology in order to identify suitable extensions to UML that are used to create our profile. The proposed profile was then applied in the modeling of a number of fragments from OBO Foundry Ontologies. The profile provides a number of UML modeling elements specific to the biomedical domain, which allows the creation of models for biomedical ontologies in a consistent and standardized way. Moreover, the use of a widely established graphical language as UML facilitates the modeling and visualization of ontologies as well as helps preventing inconsistencies usually caused by misunderstandings. Although the proposed profile can be used by any general-purpose UML modeling tool, it can also be embedded as part of specific tools in order to provide domain-specific graphical notation and/or automatic error detection.

## Methods

The following steps were used to create the OBO Relation Ontology Profile: 1) study of the OBO Relation Ontology; 2) study of the UML metamodel, UML profile mechanism and the Object Constraint Language (OCL); 3) propose an extended version of the UML metamodel in conformance with the OBO Relation Ontology; 4) propose a profile that implements the extended metamodel, and; 5) apply the proposed profile in the specification of a number of excerpts from different OBO Foundry ontologies.

### OBO Relation Ontology

The OBO Relation Ontology [33,34] defines a number of binary relations. Most of these are class-level relations defined between classes of entities (<class,class>), also known as universals, types or kinds in the literature. This ontology also includes one (primitive) relation defined between an instance and its associated class (<instance,class>). All OBO class-level relations are formally defined in terms of a set of primitive instance-level relations, connecting either two instances (<instance,instance>) or an instance to its associated class.

According to the OBO Relation Ontology, the two non-overlapping types of biological entity classes are *continuants *and *processes *[33]. *Continuants *represent things, objects, structures, while *processes *represent biological activities and events in general. The distinction between *continuants *and *processes*, also called occurrents, was first introduced in the Basic Formal Ontology (BFO) [47]. Additionally, *continuants *can be subdivided into *material *and *immaterial*. *Material *is a specific type of *continuant *that has matter, such as cell, DNA and hemoglobin. Inversely, *immaterial *is a specific type of *continuant *that has no matter, such as the interior of holes, channels, cavities and tubes.

The different relations defined in the OBO Relation Ontology are grouped into four categories: *foundational relations*, which include basic relations that are likely to be used in any biomedical ontology; *spatial relations*, which include relations that connect biological entity classes in terms of spatial regions occupied by their instances; *temporal relations*, which include relations involving biological entity classes whose instances exist at different instants of time; and, *participation relations*, which include relations between different types of biological entity classes (*processes *and participating *continuants*). Table 1 shows the different ontology relations and their respective categories.

**Table 1:** Relations of the OBO Relation Ontology

Foundational Relations	Spatial Relations	Temporal Relations	Participation Relations
instance-of			
is-a	located-in	transformation-of	
part-of	location-of	derives-from	has-participant
has-part	contained-in	derived-into	participates-in
integral-part-of	contains	preceded-by	has-agent
has-integral-part	adjacent-to	precedes	agent-in
proper-part-of			
has-proper-part			

### Unified Modeling Language

The Unified Modeling Language (UML) [44,45] is a widely disseminated language used in the modeling of computer systems. UML has been standardized by the Object Management Group [48] since the late 90 s. A major reason for wide acceptance and use of UML is the presence, in this language, of a set of semantically rich and well-defined modeling concepts. Additionally, UML is independent of specific modeling methodologies and has a wide range of support tools.

UML is defined using a metamodeling approach: a model is specified through the instantiation of model elements defined in a metamodel, i.e., a model is an instance of a metamodel. So, the main purpose of a metamodel is to define how model elements can be instantiated in a model. This metamodeling approach can be recursively applied and, thus, a model defined from a metamodel serves as a metamodel for the specification of another model. Each (meta) model represents a different layer of the (UML) metamodeling architecture.

The UML architecture is defined according to a four-layer hierarchy. The first and topmost layer, called meta-metamodel layer, UML meta-metamodel or simply M3, defines a language for the specification of metamodels. This layer contains a handful of generic modeling elements. These elements are basically grouped into a *core *package. This package is then (re)used in the specification of a number of other metamodels, including the UML metamodel itself, the Meta-Object Facility (MOF) [49] and the Profile mechanism, which since UML 2.0 has been defined as a specific metamodeling technique [44].

The second layer, called metamodel layer, UML metamodel or simply M2, consists of an instance of the meta-metamodel layer. It defines a language to describe models of an information domain. The metamodel layer represents UML itself and therefore contains the description of all UML modeling elements. It also specifies how these elements can be combined to build UML models and their associated notation.

The third layer, called model layer or M1, consists of an instance of the metamodel layer. It defines a language that describes an information domain. The model layer allows the creation of models of different interest domains, including software systems, business processes and user requirements.

Finally, the fourth and lowest layer, called object layer or M0, consists of an instance of the model layer. It comprises the (run-time) informational objects present in the interest information domain.

### UML profile

The construction of models pertaining to a particular domain can benefit from the use of more specific modeling concepts (specific syntax) than those usually provided by the language (general syntax). This can be achieved by adapting the UML metamodel in order to facilitate the construction of domain-specific models. The adaptation of the UML metamodel to a given domain provides not only specific terminology and semantics but also specific notation for the main concepts in the domain.

The adaptation of the UML metamodel can be carried out according two different approaches [44,45]. The first approach involves changing the metamodel itself, so that new elements can be added and the semantics of existing elements can be changed. The second approach extends the UML metamodel by adding constraints into existing elements of the metamodel in order to specialize their semantics. However, it is not possible to modify or contradict any original constraints related to these elements. The former type of adaptation is often called heavyweight extension and the latter is often called lightweight extension. The Profile mechanism represents a built-in mechanism for the introduction of lightweight extensions to UML.

A UML profile is a specific type of package that contains a number of extensions to the UML metamodel [44,45]. A profile must always be related to a reference metamodel; it cannot be used without its reference metamodel. A profile defines a limited capability to extend metaclasses of the reference metamodel. These extensions are defined as stereotypes that apply to existing metaclasses of the reference metamodel. A stereotype is a specific metaclass that can be used to extend an element of the reference metamodel. Thus, a stereotype defines how an existing metaclass may be extended to incorporate specific semantics and enables the use of domain-specific terminology or notation for the extended metaclass. An element of the metamodel can be extended by one or more stereotypes and, conversely, a stereotype may extend one or more elements of the UML metamodel.

The definition of a UML profile for the OBO Relation Ontology allows the elements of UML metamodel to be extended only narrowly, ensuring that the specialized metamodel is still easily understood by modelers of other domains and supported by different tools. Since a profile is a built-in mechanism of UML, it is possible to exchange profiles between UML modeling tools, as well the models to which they have been applied.

### Object constraint language

A constraint is an extensibility mechanism that allows one to refine the semantics of a modeling element or to add new semantics to this element. A constraint can also be used to limit how elements of a model can be created from elements defined in the UML metamodel. Constraints are specified via an expression written in a constraint language, either a formal language or a natural language.

Constraints specified using a natural language are intrinsically ambiguous. In order to specify unambiguous constraints a formal language must be used. The Object Constraint Language (OCL) [50] is a formal language standardized by the OMG for the description of constraints on UML models. OCL is a pure specification language. As such, the evaluation of an OCL expression can only return a value, but it cannot change the state of a model. Moreover, since OCL is not a programming language, OCL expressions can neither be directly executed nor express any implementation issue.

OCL is a typed language. Thus, each OCL expression is always associated with a type (context) represented by some element of the UML metamodel [50]. OCL expressions can be used to specify conditions that must be kept in modeling elements such as classifiers and stereotypes. Additionally, OCL expressions can be used to specify queries on UML models and describe pre and post conditions on operations and methods defined in a model, among other applications.

Constraints defined as part of the UML profile for the OBO Relation Ontology have been specified using both natural language and OCL.

## Results

### UML metamodel

**Figure 1:**
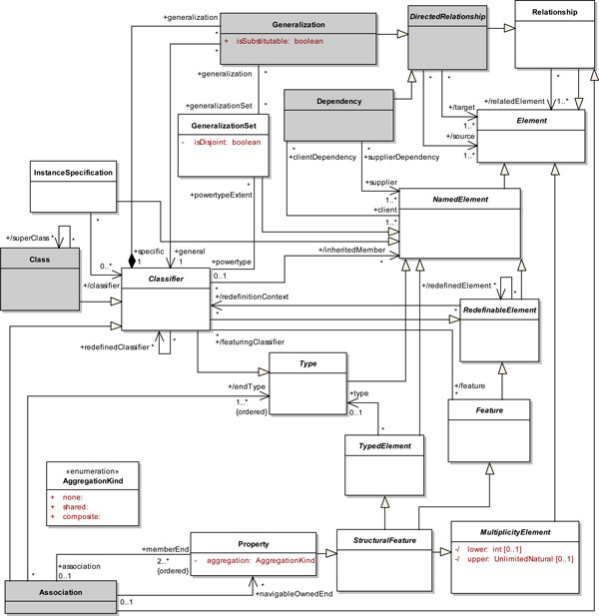
**Partial View of the UML Metamodel**. A named rectangle represents a metaclass of the UML metamodel. Abstract metaclasses have their names in italics. A gray rectangle represents an interest element for the definition of our profile. A solid line connecting two metaclasses or connecting a metaclass to itself represents an association. A filled diamond at one end of an association (aggregation end) represents a composite aggregation association. A stick arrowhead at the end of an association represents a navigable end. Finally, a line with an hollow triangle as an arrowhead connecting two metaclasses represents a generalization.

After reviewing the definitions of the different types of biological entity classes and relations of the OBO Relation Ontology, we have identified the elements in the UML metamodel that were relevant for the definition of our profile, viz., *Class*, *DirectedRelationship*, *Association*, *Generalization *and *Dependency*. Figure 1 presents an excerpt of the UML metamodel containing these elements. A number of related elements were included for completion purposes. However, we exempted ourselves from representing all existing elements and associations. More information about the UML metamodel can be found in [44,45].

The abstract metaclass *Element *represents a component of a model. *Element *is the common superclass of all metaclasses that are part of the UML metamodel. The abstract metaclass *NamedElement *specializes *Element*. *NamedElement *represents an element of a model that may have a name used to unambiguously identify this element. The abstract metaclass *TypedElement *specializes *NamedElement*. *TypedElement *represents an element that have a name and an associated type (metaclass *Type*). The abstract metaclass *Type *defines a set of values that constrains the range of values represented by a typed element. Thus, elements associated with a type are restricted to represent only values defined by the type.

The abstract metaclass *Classifier *specializes the metaclass *Type*. *Classifier *represents instances (metaclass *InstanceSpecification*) with features in common (metaclass *Feature*). *InstanceSpecification *is a named element that describes partially or completely an instance of an entity in a model. Such description can include the entity classification, i.e., the classifier(s) from which the entity is an instance, and, based on its classifier, the kind of instance (e.g. object or link). *InstanceSpecification *can also be used to represent a snapshot of an existing entity at some point in time. The abstract metaclass *Feature *is a named element that represents behavioral or structural characteristics of classifiers.

The metaclass *Relationship *specializes the metaclass *Element. Relationship *represents a type of relation between two or more elements of a model. The abstract metaclass *DirectedRelationship *specializes *Relationship *in order to represent directed relations between source and target elements. The metaclass *Dependency *specializes *DirectedRelationship*. *Dependency *represents a relation defined between named elements of a model in which a set of (*client*) elements require other elements (*supplier*) for their (complete) specification. This relation establishes that the semantics of the *client *element(s) is dependent on the definition of the *supplier *element(s).

The metaclass *Generalization *also specializes *DirectedRelationship*. *Generalization *represents a binary relation between a general classifier and a more specific classifier. This relation is used to represent that instances of the specific classifier are also instances of the general classifier. Thus, any feature defined for the general classifier is inherited by the specific classifier. Similarly, any constraint applied to the general classifier is also applied to the specific classifier. *Generalization *has one boolean attribute, *isSubstitutable *(default value is *true*), which indicates whether or not the specific classifier can be used wherever the general classifier is usually used.

The abstract metaclass *RedefinableElement *specializes the abstract metaclass *NamedElement*. *RedefinableElement *represents an element that can be redefined in the context of a generalization. Since *Classifier *and *Feature *are specializations of *RedefinableElement*, they can be redefined in the context of a generalization relation. The redefinition of an element can include semantics addition or restriction in a manner consistent with the semantics initially defined.

Generalization relations can be aggregated into subsets. The metaclass *GeneralizationSet *is a named element that represents collections of subsets of generalization relationships. *GeneralizationSet *describes how a single general classifier (powertype) can be subdivided into several specific subtypes. *GeneralizationSet *has two boolean attributes, viz., *isCovering *(default value is *false*), which indicates whether or not every instance of a general classifier is also an instance of at least one of its specific classifiers, and *isDisjoint *(default value is *false*), which indicates whether or not the set of specific classifiers in a generalization have an instance in common.

The abstract metaclass *MultiplicityElement *specializes the abstract metaclass *Element*.*MultiplicityElement *defines an inclusive interval of non-negative integers beginning with a lower bound, attribute *lower *(default value is *one*), and ending with a possibly infinite upper bound, attribute upper (default value is also *one*). *MultiplicityElement *specifies the allowable cardinalities for an instantiation of this element. The abstract metaclass *StructuralFeature *specializes the metaclasses *Feature*, *TypedElement *and *MultiplicityElement*. *StructuralFeature *represents a typed feature of a classifier that specifies the structure of instances of the classifier. The metaclass *Property *specializes the metaclass *StructuralFeature*. In the context of this work, *Property *represents the types of association ends.

The metaclass *Association *specializes the metaclasses *Classifier *and *Relationship*. *Association *represents a semantic relationship that can occur between instances of typed elements. *Association *instances are named links. An association has at least two (ordered) ends (*memberEnd*), each one represented by a property and indirectly associated to a corresponding type (*endType*). A member end represents the participation of an instance of the classifier connected to an end of a link. Thus, an association declares that there can be links between instances of associated types. Additionally, an association may have one or more navigable ends (*navigableOwnedEnd*). A navigable end can be more easily accessed at runtime from instances participating in the other end(s) of the link. Navigable ends provide a navigation facility.

Aggregation represents a specific type of binary association in which elements representing "parts" are related to an element representing a "whole" (whole/part relationship). Two different types of aggregation can be defined, viz., composition and shared aggregation. Composition represents an aggregation relation in which instances of "part" can only be included in a single composition. Additionally, if the instance representing the "whole" is removed, the parts are removed as well. A shared aggregation poses no such restriction. In such relation, instances of "part" can be included in more than one shared aggregation. Further, if an instance representing the "whole" is removed, the parts may or may not be removed. Both relationships are represented in the UML metamodel by the attribute *aggregation*, whose type is *AggregationKind*. *AggregationKind *is an enumeration type that represents different types of association: association without aggregation (*none*), association with composition (*composite*) and association with shared aggregation (*shared*).

The metaclass *Class *specializes the metaclass *Classifier*. *Class *describes a set of instances (objects) that share features, constraints and semantics. The structural and behavioral features owned by a class (not depicted in Figure 1) are named attributes and operations, respectively. Objects of a class have their own values for attributes. These values are in accordance with the types and multiplicities defined by the class. Operations defined for a class can be invoked on objects of the class. As a result, the invocation of an operation on an object can return a value and/or cause changes in the values of attributes of this object. In addition, operation invocation can also cause changes in the values of attributes of other objects that can be reached through the links associated to the object on which the operation was invoked.

### UML profile for the OBO Relation Ontology

In order to define our profile for the OBO Relation Ontology, we have proposed a number of extensions for the UML metamodel. These extensions were proposed based on the definitions of the types of biological entity classes and relations of the OBO Relation Ontology.

The different types of biological entity classes defined in the OBO Relation Ontology were represented as specializations of metaclass *Class*. The metaclass *Class *was initially specialized into the metaclasses *Continuant *and *Process*. The metaclass *Continuant *was in turn also specialized into the metaclasses *Material *and *Immaterial*. By default, these metaclasses (*Continuant/Process *and *Material/Immaterial*) are mutually exclusives. These definitions are consistent with the principles of the OBO Relation Ontology that describes these categories as non-overlapping. Thus, classes extended by *Process *can not be extended by *Continuant *or its subtypes and vice versa, and classes extended by *Material *can not be extended by *Immaterial *and vice versa.

Each of the proposed extension elements corresponds to a stereotype in our profile. Thus, four stereotypes were defined for representing the type of a biological entity class, viz., <<*continuant*>>, <<*process*>>, <<*material*>> and <<*immaterial*>>.

**Figure 2:**
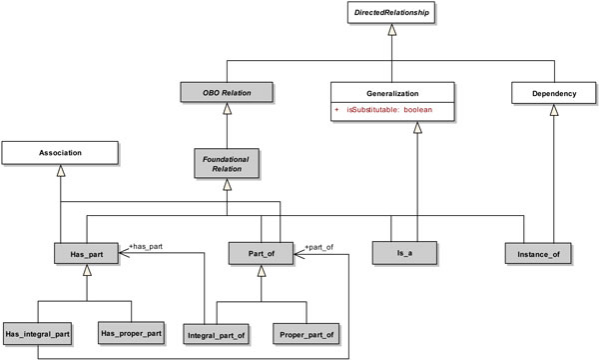
**Foundation Relations Extensions**. A white rectangle represents an original metamodel element, while a gray rectangle represents an extension element.

Figure 2 depicts the extensions proposed to the UML metamodel to capture the different foundational relations of the OBO Relation Ontology. The different types of relations are specializations of abstract metaclass *OBORelation*, which in turn specializes the abstract metaclass *DirectedRelationship*. *OBORelation *represents directed and binary (between two classes) relations that may occur between continuants, including material and immaterial continuants, and processes.

The abstract metaclass *FoundationalRelation *represents basic relations that can be defined between two continuant entity classes or between two process entity classes. The metaclass *Is_a *specializes the abstract metaclass *FoundationalRelation*. *Is_a *represents a subtype relation between a biological entity class (source) and another biological entity class (target) acting as a supertype. Since the metaclass *Generalization *defines a similar type of relationship, *Is_a *also specializes *Generalization*.

The metaclass *Instance_of *specializes the abstract metaclass *FoundationalRelation*. *Instance_of *represents a primitive relation between a general biological entity class (*continuant *or *process*) and a particular instance of this class (instance-class relation). Since the UML metaclass *Dependency *represents a relationship that can occur between named elements in general, such that a set of client elements is either semantically or structurally dependent on the definition of a set of supplier elements, we have used this metaclass as basis for the representation of *Instance_of*. Thus, *Instance_of *was also defined as a specialization of *Dependency*.

The metaclasses *Part_of *and *Has_part *also specialize the metaclasses *FoundationalRelation *and *Association*. *Part_of *represents an association between a source and a target biological entity class, in which each instance of the source class is part of an instance of the target class (whole). Inversely, *Has_part *represents an association between a source biological entity class and a target biological entity class, in which an instance of the source class (whole) has other instances of the target class as its parts.

*Part_of *is specialized into the metaclasses *Proper*_*Part_of *and *Integral*_*part_of*. *Proper*_*part_of *represents a *Part_of *relation with the additional constraint that the source entity class is different than the target entity class (*Part_of *has not such constraint). Additionally, in a *Part_of *relation defined between a source entity class and a target entity class, we cannot infer that the target has the source as its part. Such semantics is captured by the *Integral_part_of *relation. *Integral*_*part_of *represents a *Part_of *relation in which the target entity class has also the source entity class as its part (represented by the association *has_part*).

Similarly, *Has_part *is also specialized into two metaclasses, viz., *Has_proper_part *and *Has_integral_part*. *Has_proper_part *represents a *Has_part *relation with the additional constraint that the source entity class is different than the target entity class. *Has_integral_part *represents a *Has_part *relation in which the target entity class is also part of the source entity class (represented by the association *part_of*).

Each of the proposed extension elements corresponds to a concrete stereotype in our profile, except for the abstract metaclasses *OBORelation *and *FoundationalRelation*. Thus, the following stereotypes were defined for representing a foundation relation: <<*is_a*>>, <<*instance_of*>>, <<*part_of*>>, <<*integral*_*part_of*>>, <<*proper*_*part_of*>>, <<*has_part*>>, <<*has_integral_part*>> and <<*has_proper_part*>>.

The *Is_a *relation (*C Is_a C*_1_) is formally defined as follows: if *c *instantiates *C *at a time *t*, then *c *instantiates *C*_1 _at *t*, where both *C *and *C*_1 _represent either *continuant *or *process *entity classes. The UML metaclass *Generalization*, which we have used as basis for the definition of *Is_a*, represents a relationship that can occur between one specific classifier and one general classifier, such that an instance of the specific classifier is also an instance of the general classifier. Provided the specific and the general classifiers represent either two *continuant *entity classes or two *process *entity classes, we are able to capture in UML a semantic definition equivalent to the one defined in the OBO Relation Ontology for this relation. These restrictions have been defined as part of <<*is_a*>> stereotype specification.

The *Instance_of *relation (*c Instance_of C*) represents a primitive relation between an instance *c *and an entity class *C*, either *continuant *or *process*, which it instantiates at a specific time *t*. The UML metaclass *Dependency*, which we have used as basis for the definition of *Instance_of*, represents a relationship in which one or more named elements (*client*) are dependent on the definition of one or more named elements (*supplier*). Provided the client represents a particular instance of an entity class (*InstanceSpecification*) and the supplier represents the entity class itself, either *continuant *or *process*, which it instantiates, we are able to capture in UML a semantic definition equivalent to the one defined in the OBO Relation Ontology for this relation. These restrictions have been defined as part of <<*instance_of*>> stereotype specification.

The *Part_of *relation (*C Part_of C*_1_) is formally defined as follows: for all *c *that instantiates *C *at a time *t*, there is some *c*_1 _such that *c*_1 _instantiates *C*_1 _at time *t *and *c ***part_of ***c*_1 _at *t*, where both *C *and *C*_1 _represent either *continuant *or *process *entity classes and **part_of **represents a primitive instance-level relation. The all/some rule used in the definition of the *Part_of *relation guarantees that this relation is valid for every instance of class *C *being related to some instance of class *C*_1_.

The UML metaclass *Association*, which we have used as basis for the definition of *Part_of*, models the existence of a semantic relationship (link) between instances of typed elements. A link is an instance of an association. In order to relate all instances of class *C *to at least one instance of class *C*_1 _through links, we have constrained the <<*part_of *>> stereotype using the *forAll *and *exists *OCL operators. However, the pivotal difference between the OBO Relation Ontology and UML lies in the fact that instance-level relations are formally defined in the former, i.e., they form a set of primitive relations, whereas links are not formally defined in the latter. In this regard, our profile falls short in representing exactly the same semantics as defined by the OBO Relation Ontology for the *Part_of *relation.

The approach used to capture the semantics of the <<*part_of*>> stereotype has also been used in the specification of the remaining stereotypes of the profile because their corresponding OBO relations have also been formally defined using the *all/some *rule and, similarly to <<*part_of*>>, they also specialize the metaclass *Association*.

**Figure 3:**
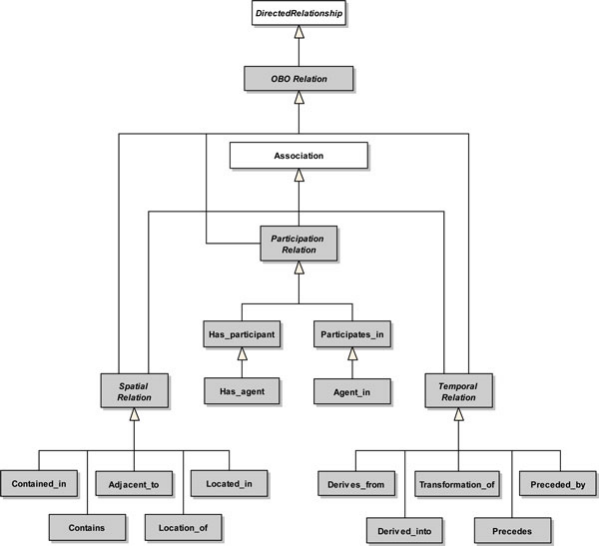
**Participation, Spatial and Temporal Relations Extensions**. A white rectangle represents an original metamodel element, while a gray rectangle represents an extension element.

Figure 3 depicts the extensions proposed to the UML metamodel to capture temporal, spatial and participation relations of the OBO Relation Ontology.

The abstract metaclass *SpatialRelation *represents spatial relations defined between different continuant entity classes. *SpatialRelation *specializes the metaclasses *OBORelation *and *Association*. The metaclasses *Adjacent_to*, *Located_in*, *Location_of*, *Contained_in *and *Contains *are all specializations of *SpatialRelation*.

*Adjacent_to *represents that the spatial region occupied by a source continuant is adjacent to the spatial region occupied by a target continuant (no overlapping). *Located_in *represents that a source continuant is located in the spatial region occupied by a target continuant. *Contained_in *represents that a source material continuant is contained in the spatial region occupied by a target immaterial continuant. However, in this case, the material continuant is not part of the immaterial continuant. *Location_of *and *Contains *represent the inverse of relations *Located_in *and *Contained_in*, respectively.

Each of the proposed extension elements corresponds to a concrete stereotype in our profile, except for the abstract metaclass *SpatialRelation*, which is also used to aggregate common properties of its subtypes and help structuring the profile. Thus, the following stereotypes were defined for representing a spatial relation: <<*adjacent_to*>>, <<*located_in*>>, <<*location_of *>>, <<*contained_in*>> and <<*contains*>>.

The abstract metaclass *TemporalRelation *represents temporal relations defined between different entity classes. *TemporalRelation *specializes the metaclasses *OBORelation *and *Association*. The metaclasses *Derives_from*, *Derived_into*, *Transformation_of*, *Preceded_by *and *Precedes *are all specializations of *TemporalRelation*.

*Derives_from *represents that a source material continuant immediately derives from a target material continuant. The target continuant ceases to exist and (part of) its matter is inherited by the source continuant. *Transformation_of *represents that a source material continuant results from the transformation of a target material continuant (target continuant instantiates the source continuant). *Preceded_by *represents that a target process occurs in an instant of time prior to the occurrence of a source process. *Derived_into *and *Precedes *represent the inverse of relations *Derives_from *and *Preceded_by*, respectively.

Each of the proposed extension elements corresponds to a concrete stereotype in our profile, except for the abstract metaclass *TemporalRelation*, which is also used to aggregate common properties of its subtypes and help structuring the profile. Thus, the following stereotypes were defined for representing a temporal relation: <<*derives_from*>>, <<*derived_into*>>, <<*transformation_of *>>, <<*preceded_by*>> and <<*precedes*>>.

Finally, the abstract metaclass *ParticipationRelation *represents participation relations of continuants in the occurrence of processes. *ParticipationRelation *also specializes the metaclasses *OBORelation *and *Association*. The metaclass *Has_participant *specializes the metaclass *ParticipationRelation*. *Has_participant *represents that a target continuant participates somehow in a source process. *Has_agent *specializes the metaclass *Has_participant*. *Has_agent *represents that a source process has a material continuant as its participant and that this continuant is responsible for the occurrence of the process. *Participates*_*in *and *Agent_in *represent the inverse of relations *Has_participant *and *Has_agent*, respectively.

Each of the proposed extension elements corresponds to a concrete stereotype in our profile, except for the abstract metaclass *ParticipationRelation*. Thus, the following stereotypes were defined for representing a participation relation: <<*has_participant*>>, <<*participates_in*>>, <<*has_agent*>> and <<*agent_in*>>.

**Figure 4:**
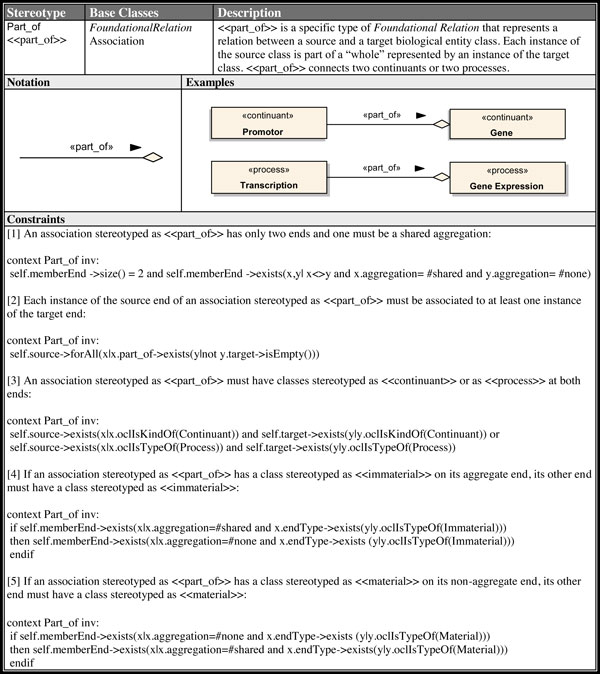
****<<**part_of**>>**Stereotype Definition**.

The abstract metaclasses *OBORelation*, *FoundationalRelation*, *SpatialRelation, TemporalRelation *and *ParticipationRelation *were introduced to aggregate common properties of its subtypes and help structuring the profile. Thus, we did not define a concrete syntax for these metaclasses in our profile. For each element defined in our profile, there is a brief description of its semantics, the base class(es) extended by the stereotype, associated notation and at least one example of its usage. Additionally, we also described any constraints that must be applied to elements extended by these stereotypes. These constraints were described using both text and an equivalent OCL expression. Figure 4 illustrates an example of a profile element definition (<<*part_of *>> stereotype).

**Figure 5:**
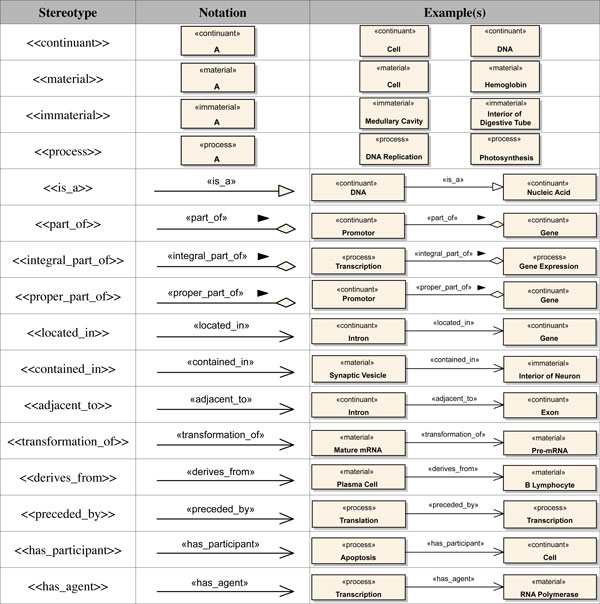
**Summary of the OBO Relation Ontology Profile Specification**.

Figure 5 shows a summary of selected stereotypes in terms of corresponding notation and example(s) of usage. Note that the stereotypes defined for the different types of entity classes have a notation similar to UML classes, while the <<*is_a*>> stereotype presents a notation similar to a UML generalization. Finally, the <<*part_of *>> stereotype and its subtypes present a notation similar to a UML shared aggregation. We have chosen the notation similar to shared aggregation instead of composition because the former is less restrictive than the latter. All other stereotypes have a notation similar to UML associations. The complete profile specification can be found in a supplementary material (see Additional File 1).

### Profile application

This section describes the application of the proposed profile in the development of a number of fragments from different (standard) ontologies. The objective of this activity was to evaluate the use of the profile in the specification of a number of UML models. We have focused only on OBO Foundry ontologies. Particularly, we have considered the following ontologies: Gene Ontology (GO), PRotein Ontology (PRO) and Xenopus Anatomy and Development Ontology (XAO). Thus, no OBO Foundry candidate ontologies and/or other ontologies of interest were considered. Additionally, since the relationships defined in the OBO Relation Ontology represent the vast majority of the total relationships defined on these ontologies (over 90% in some cases), the fragments were chosen to focus only on these relationships. We have used Enterprise Architect, from Sparx Systems, as our UML modeling tool.

The first ontology considered in our study was the Gene Ontology (GO) [3]. GO provides a set of terms and relations used for standardization of genes and their products in eukaryotic organisms using three independent ontologies: *Cellular Component*, which describes subcellular structures and macromolecular complexes in which, generally, gene products can be located in or can be subcomponents of; *Molecular Function*, which describes activities that occur at the molecular level; and *Biological Process*, which describes collections of processes (series of events or molecular functions) related to the functioning of integrated living units. In the context of our work, we have considered only the *Cellular Component *ontology. In the fragments considered in the development of our models, only continuants were identified. Examples of these continuants include *Cell Part*, *Cell Body*, *Membrane*, etc. *Is_a *and *Part_of*, which account for over 92% of the total relationships defined by GO, represent the only relationships used in these fragments.

The second ontology considered in our study was the PRotein Ontology (PRO) [27]. PRO has been developed by the National Institute of General Medical Sciences (NIGMS) to describe proteins (protein forms) and protein evolutionary relationships (protein evolution). Thus, PRO has two overlapping components: *Protein Evolution (ProEvo) *and *Protein Forms (ProForm)*. *ProEvo *organizes proteins according to their evolutionary relatedness, while *ProForm *describes multiple proteins forms derived from a given gene, which arise through variations in splicing or post-translational modifications.

Each concept represented by the ontology has a unique identifier within the scope of its components. Additionally, multiple protein forms produced from a given gene are referred as isoforms, and polymorphic sequences as variants. In the fragments considered in the development of our models, only (material) continuants were identified. *Is_a *and *Derives_from*, which account for over 90% of the total relationships defined on the PRO, represent the only relationships used in these fragments. In particular, *Derives_from *is used to indicate proteins with post-translational modifications derived from non-modified proteins.

**Figure 6:**
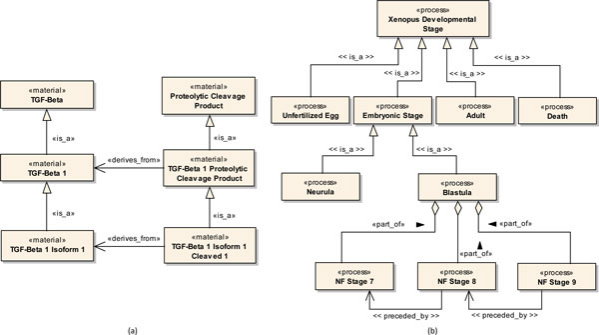
**Profile Application**. (a) Fragment of the PRotein Ontology. (b) Fragment of the Xenopus Anatomy and Development Ontology.

Figure 6a illustrates a modeled fragment of the PRO. All depicted elements represent proteins. Since proteins refer to entity classes that have molecular weight, they are considered material continuants (<<*material*>>) according to the OBO Relation Ontology.

The class *TGF-Beta *represents a protein involved in the regulation of cell growth and differentiation. The class *TGF-Beta 1 *represents a TGF-beta protein that is a translation product of the TGFB1 gene. Thus, it was modeled as a specialization of the class *TGF-Beta *through an *Is_a *relation (<<*is_a*>>). The class *Proteolytic Cleavage Product *represents an amino acid chain produced as the result of peptide bond cleavage of a longer amino acid chain. The class *TGF-Beta 1 Proteolytic Cleavage Product *represents a proteolytic cleavage product that is derived from TGF-beta 1 protein. Thus, it was modeled as a specialization of *Proteolytic Cleavage Product *through an *Is_a *relation. Additionally, a *Derives_from *relation (<<*derives_from*>>) was also established between this class and the class *TGF-Beta 1*.

The class *TGF-Beta 1 Isoform 1 *represents a translational product of a specific transcript of the TGFB1 gene. Thus, it was modeled as a specialization of the class *TGF-Beta 1 *through an *Is_a *relation. The class *TGF-Beta 1 Isoform 1 Cleaved 1 *represents a specific product of TGF-Beta 1, which was modified by a specific proteolytic cleavage process. The class *TGF-Beta 1 Isoform 1 Cleaved 1 *represents a TGF-beta 1 proteolytic cleavage product that is derived from a TGF-beta 1 isoform 1 protein that suffered a proteolytic cleavage process. Thus, it was modeled as a specialization of *TGF-Beta 1 Proteolytic Cleavage Product *through an *Is_a *relation. Additionally, a *Derives_from *relation (<<*derives_from*>>) was also established between this class and the class *TGF-Beta 1 Isoform 1*.

The third ontology considered in our study was the Xenopus Anatomy and Development Ontology (XAO) [28]. XAO was created to standardize the annotation of gene expression, normal and mutant phenotypes data of Xenopus species. This ontology has two overlapping components, viz., *Xenopus Anatomical Entity *and *Xenopus Developmental Stage*. The former provides a description of anatomical structures and tissues of the specie and the latter provides a description of the developmental stages of the specie. Each concept represented by the ontology has a unique identifier within the scope of these two components. In the context of our work, we have considered only the *Xenopus Developmental Stage *ontology. In the fragments considered in the development of our models, only processes were identified. *Is_a*, *Part_of *and *Preceded_by*, which account for over 63% of the total relationships defined on the XAO, represent the only relationships used in these fragments. In particular, *Preceded_by *is used between developmental stages with the purpose of indicating time intervals during which certain anatomical structures and tissues exist.

Figure 6b illustrates a modeled fragment of the XAO. All depicted elements represent developmental stages of Xenopus species. Since stages refer to entity classes that have a beginning, middle and end, they are considered processes according to the OBO Relation Ontology.

The class *Xenopus Developmental Stage *represents any developmental stage of the Xenopus species. Classes *Unfertilized Egg*, *Embryonic Stage*, *Adult *and *Death *represent different developmental stages of this organism, each modeled as a specialization of *Xenopus Developmental Stage *through an *Is_a *relation (<<*is_a*>>).

The class *Embryonic Stage *represents a developmental stage that occurs in the time interval between fertilization and body feeding. The classes *Blastula *and *Neurula *represent specific embryonic developmental stages that occur within this time interval and thus they were modeled as specializations of *Embryonic Stage *through *Is_a *relations.

*Blastula *comprehends a range of developmental stages that occur between the Nieuwkoop and Faber (NF) stage 7 and NF stage 9. Each of these stages was modeled as a separate class, viz., *NF Stage 7*, *NF Stage 8 *and *NF Stage 9*. *NF Stage 7 *represents a four hour 64-cell embryo. *NF Stage 8 *represents a five hour 128-cell embryo. *NF Stage 9 *represents a seven hour embryo whose cells are smaller at dorsal than at ventral side. Since these classes are part of the range defined by *Blastula*, each was related to *Blastula *through a *Part_of *relation (<<*part_of *>>).

Classes *NF Stage 8 *(source) and *NF Stage 7 *(target) were related through a *Preceded_by *relation (<<*preceded_by*>>). This same type of relation was defined between classes *NF Stage 9 *and *NF Stage 8*. Since stages NF stage 7, NF stage 8 and NF stage 9 happen in Xenopus species respectively at 4, 5 and 7 hours (22-24 °C) after the embryo fertilization, the application of this relation was consistent with its definition because NF stage 7 occurs in an instant of time preceding NF stage 8 and likewise NF stage 8 occurs in an instant of time preceding NF stage 9.

## Discussion

We have developed a UML profile for the OBO Relation Ontology. First, we have proposed a number of extensions to the UML metamodel in conformance with the OBO Relation Ontology. Then, these extensions were mapped onto corresponding elements of our profile. Finally, we have modeled a number of fragments of OBO Foundry ontologies using the profile. Due to the graphical domain-specific modeling elements of the proposed profile, the modeling and visualization of ontologies in the biomedical domain was facilitated. Additionally, the profile provided support for more intuitive forms of reasoning about the incorporation of elements into ontologies than text-based formats.

UML has increasingly been used for the representation of biomedical knowledge [51-55]. According to these works, UML graphical notation enables the representation of complex biological data and allows biologists to visualize and interpret information in an intuitive way. UML also improves the description of the semantics of a given domain. These works also highlight the use of UML intrinsic extension mechanism to tailor the language to particular biological purposes.

Due to the ontological commitments of UML, we were not able to formally represent, for most of the relations in our profile, exactly the same semantics as defined by the OBO Relation Ontology. This limitation is not a problem because the profile should not be used in isolation, but having in mind the formal definitions of the OBO Relation Ontology.

The developed UML profile is useful for modeling and visualizing ontologies, but no complete ontology was modeled, i.e., only fragments of selected biomedical ontologies were modeled using the profile. Biomedical ontologies are in general large artifacts, which poses a challenge regarding its representation and visualization using a graphical notation and supporting tool. Actually, any large model presents the same limitation. However, this limitation can be overcome with adequate support from a modeling tool (including zooming functionalities). Additionally, the application of the profile aimed primarily at demonstrating its use to model different sets of biomedical entity classes and their relationships. Since, for example, *Is_a *and *Part_of *represent over 90% of the GO relationships and the remaining 10% of its relationships do not pertain to the OBO Relation Ontology, it is needless to model the complete Gene Ontology for this purpose.

Ontology developers and users can benefit from a well-established language such as UML. Even though ontology developers are more likely to know UML, most ontology users in the biomedical domain, biologists in general, are less likely to know this language. However, the UML graphical notation is as difficult to learn as any other graphical notation. Additionally, even in the biomedical domain UML graphical notation can be considered quite intuitive and easy to learn [51,54].

Continuous modifications of existing ontologies according to emerging new biological insights represent a common practice in the biomedical domain. Thus, any standards proposed to represent (graphically or not) biomedical ontologies must be flexible enough to, possibly, accommodate these changes. Recently, the OBO Foundry started developing a new version of the OBO Relation Ontology [56] to replace the current ontology. The new OBO Relation Ontology includes a number of changes, such as the inclusion of some biomedical-specific relations and the restructuring of the hierarchy of entity classes. Since this new version has been neither finalized (most relations presents a "pending final vetting" curation status) nor used in the development of any OBO ontology, the ontology which was used as basis for our work remains the *de facto *standard.

Eventually, the new version of the OBO Relation Ontology will become the *de facto *standard and the developed UML profile will need to be restructured to incorporate the proposed modifications. Basically, we will need to add and/or replace a number of abstract and concrete metaclasses to reflect both the new hierarchy of entity classes and the updated list of relations. Then, these elements will be mapped to corresponding stereotypes in the profile. We believe our UML profile is flexible enough to accomodate these modifications in due time.

To the best of our knowledge this is the first initiative to provide a formal conceptual framework to support the UML-based development of ontologies according to OBO principles. UML graphical notation facilitates the visualization of concepts and their relationships, which helps preventing inconsistencies frequently introduced during the incorporation of new concepts and/or relationships to an ontology. Additionally, since the profile concepts are formally defined using OCL, their use can be subject to (automatic) reasoning which also helps preventing inconsistencies.

According to the OBO Relation Ontology, a biological entity class is classified as either continuant or process. Additionally, a continuant can be further classified as either material or immaterial. However, OBO Foundry ontologies do not make such distinctions. Concepts are included in the ontology without any explicit reference or association to this ontological classification. Our UML profile, by contrast, requires each modeled entity class to be stereotyped as either <<*continuant*>> or <<*process*>>. Additionally, continuants can also be stereotyped as either <<*continuant,material*>> or <<*continuant*,*immaterial*>> to indicate a more detailed level of classification. However, in this case the <<*material*>> and <<*immaterial*>> stereotypes can simply be used instead.

The sole association of a concept to the different types of entity classes helps preventing the introduction of inconsistencies and/or facilitates the identification of existing inconsistencies in developed ontologies. Consider, for example, the concept *Gene Ontology*, which was initially defined in the Gene Ontology to represent the ontology itself. According to the profile definition, the concept *Gene Ontology *could not have been stereotyped neither as <<*continuant*>> nor as <<*process*>>. Thus, characterizing an invalid concept.

Additionally, since the concepts contained in OBO Foundry ontologies are not explicitly classified, relationships between these concepts are also established based only on tacit knowledge. Although ontology development is carried out largely by domain experts, such reliance on tacit knowledge favours the arising of (semantics) inconsistencies. On the other hand, since each modeled entity class using our profile is explicitly stereotyped, any relation established between them is subject to the (formally) defined ontological constraints of this relation. For example, the relation *Has_agent *connects a source process to a target material continuant. So, any attempt to connect other types of entity classes, e.g., a source process to a target immaterial continuant or two material continuants, using this relation is incorrect. The use of our profile allows ontology developers and ontology users to explicitly reason about these constraints. Furthermore, in case automatic support is provided by the modeling tool for integrity check, an error message can be generated or the addition of the relation can be prevented at all (see, for example, [57]), thus assuring the accuracy of the model under development.

Considering once more the previous example of the Gene Ontology, the concepts *Biological Process*, *Cellular Component *and *Molecular Function *were all related to the *Gene Ontology *concept through separate *Is_a *relations to indicate these concepts were terminological components of the Gene Ontology [58]. Since *Gene Ontology *could not have been stereotyped neither as <<*continuant*>> nor as <<*process*>>, the *Is_a *relation defined, for example, between *Biological Process *and *Gene Ontology *would have been clearly invalid because an *Is_a *relation can only be used to connect either two continuants or two processes (another indication of inconsistency). All these inconsistencies have been removed from the Gene Ontology since the exclusion of the *Gene Ontology *concept.

**Figure 7:**
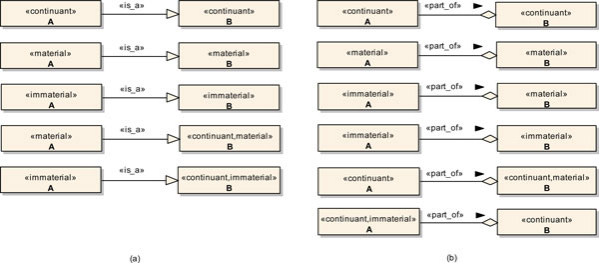
**Application of the Profile in the Creation of Valid Is_a and Part_of Relations Between Generic Continuants**. (a) *Is_a *relations. (b) *Part_of *relations.

Figure 7 illustrates the application of the profile constraints in the creation of different sets of models relating two generic continuants *A *and *B *through *Is_a *(Figure 7a) and *Part_of *relations (Figure 7b), which represent two of the most important relations defined in the OBO Relation Ontology.

Basically, we can always relate two unspecified source and target continuants, i.e., two entity classes stereotyped only as <<*continuant*>>, through an *Is_a *relation, provided there is semantic soundness in the association. However, once the source or the target continuant has been stereotyped either as <<*material*>> or as <<*immaterial*>>, the other continuant has to be properly stereotyped as well. So, we can only relate a source material continuant to a target material continuant, while we can only relate a source immaterial continuant to a target immaterial continuant.

Similarly, we can always relate two unspecified source and target continuants through a *Part_of *relation, provided there is semantic soundness in the association as well. However, once the source continuant has been stereotyped as <<*material*>>, the target continuant has to be stereotyped as <<*material*>> as well. Additionally, once the target continuant has been stereotyped as <<*immaterial*>>, the source continuant has to be stereotyped as <<*immaterial*>> as well. So, a source material continuant can only be part of a target material continuant, while a target immaterial continuant can only have a source immaterial continuant as its part. Nevertheless, a source immaterial continuant can be part of either an immaterial or a material continuant.

The use of the UML profile also improves the readability of developed ontologies. Consider, for example, multiple inheritance in biomedical ontologies, i.e., a single source entity class specializing multiple target entity classes through separate *Is_a *relations. On one hand, multiple inheritance facilitates the creation of compact and easily navigable models. On the other hand, multiple inheritance poses a problem to the (automatic) integration of different biomedical ontologies (concept alignment) due to the assignment of multiple meanings to the same relation within a single ontology [59]. Multiple inheritance can be more easily spotted and understood using graphical models and, in many cases, it can be avoided by replacing one (or more) *Is_a *relation(s) by other types of relations, thus eliminating this problem.

**Figure 8:**
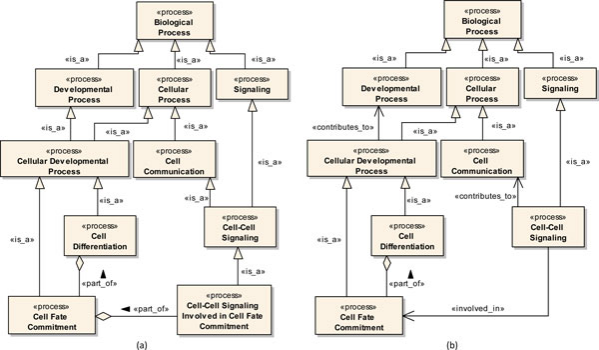
**Gene Ontology Fragment**. (a) Original fragment (version August 2011). (b) Fragment with proposed modifications.

Figure 8 illustrates an example of multiple inheritance in the Gene Ontology. According to this fragment, the class *Cellular Developmental Process *specializes both *Developmental Process *and *Cellular Process *(Figure 8a). In this case, the *Is_a *relation between *Cellular Developmental Process *and *Developmental Process *can be more properly expressed as a *Contributes_to *relation (contributes to the achievement of a certain end) [59], thus eliminating the multiple inheritance. Similarly, the class *Cell-Cell Signaling *specializes both *Cell Communication *and *Signaling*. In this case, the use of the *Contributes_to *relation between *Cell-Cell Signaling *and *Cell Communication *can also be more appropriate. Figure 8b shows these proposed modifications to the modeled GO fragment.

As a final example of how the proposed profile can be used to improve an existing ontology, consider the class *Cell-Cell Signaling Involved in Cell Fate Commitment*, also depicted in Figure 8. According to the current version of the Gene Ontology (August 2011), this concept both specializes *Cell-Cell Signaling *and is part of *Cell Fate Commitment*. However, instead of using *Cell-Cell Signaling Involved in Cell Fate Commitment *to indicate a relation (involved in) between *Cell-Cell Signaling *and *Cell Fate Commitment*, it would be more appropriate to directly relate these two classes, using, for example, a relation *Involved_in*, thus improving the quality of the ontology in general. Other examples of concepts from the Gene Ontology representing the same type of relation include *MAPKKK Cascade Involved in Osmosensory Signaling Pathway*, *Wnt Receptor Signaling Pathway Involved in Mammary Gland Specification *and *Proteolysis Involved in Cellular Protein Catabolic Process*.

Finally, we believe our UML profile can also be used in combination with model-driven approaches to adequately capture biological knowledge and promote fast system development. Model-Driven Architecture (MDA) represents an approach to system development proposed by OMG [60]. According to this approach, models are used throughout the software life cycle, including understanding, design, construction, deployment, operation, maintenance and modification of software systems. System models are developed according to different viewpoints or perspectives and support is provided for the (successive) transformation of one (more abstract) model into another (more concrete) model until an implementation in a specific platform is obtained. MDA has attracted increasing interest from the biomedical community [61-63]. We believe our profile can be used to support the development of models according to all defined MDA viewpoints, but mainly computational independent and platform independent viewpoints.

## Conclusions

Ontologies are increasingly being used in the biomedical domain. Not only new ontologies are being developed for new domains, but also existing ontologies are continuously being improved due to the efforts of a growing number of developers and users. Initiatives to facilitate the development and integration of (biomedical) ontologies have emerged. In this sense, the OBO Foundry provides a repository of life-science ontologies developed according to a set of shared principles. The OBO Foundry has also developed the OBO Relation Ontology to allow the standardization of different types of entity classes and associated relationships in the biomedical domain.

Correctness is key for the success of any ontology. In this sense, we have developed a UML profile for the OBO Relation Ontology to support the development of UML-based models of biomedical ontologies in a consistent and standardized way, hence contributing for ontology correctness and accuracy. Our results indicate that the use of the profile requires the domain expert to reason about the underlying semantics of the concepts and relationships being modeled, thus preventing the introduction of inconsistencies in an ontology under development and facilitating the identification and correction of errors in an existing ontology.

Future research is needed towards the development of adequate tool support for the UML profile for the OBO Relation Ontology. Although one can use the profile in any general-purpose UML modeling tool, biologists can benefit from the development of domain-specific tools that support not only the visualization and editing of models, but also automatic processing of integrity rules representing defined ontological constraints over produced models. Similarly to the translation of ontologies in OBO format to OWL [64], tools can be initially developed to transform UML models developed according to the profile into OBO format, possibly using a set of tags to represent additional semantics. Later, other tools can be developed to transform ontologies in either OBO format or OWL into UML using, for example, the XML Metadata Interchange (XMI) format [65], thus contributing to unfold the profile full potential.

## Competing interests

The authors declare that they have no competing interests.

## Authors' contributions

GG defined and applied the profile and drafted the manuscript. RV participated in the profile application and participated in the drafting of the manuscript. CF defined and applied the profile and drafted the manuscript. All authors read and approved the final manuscript.

## Supplementary Material

Additional File 1 **Specification of the UML Profile for the OBO Relation Ontology**. Detailed description of the stereotypes and metaclasses defined in UML profile for the OBO Relation Ontology. Click here for file
