# Supplementary material for: A UML profile for the OBO relation ontology
Source: BMC Genomics. 2012 Oct 19;13(Suppl 5):S3. doi: 10.1186/1471-2164-13-S5-S3 (PMC3477006; doi:10.1186/1471-2164-13-S5-S3)
Supplement: Additional File 1 — Specification of the UML Profile for the OBO Relation Ontology. Detailed description of the stereotypes and metaclasses defined in UML profile for the OBO Relation Ontology. [file 1471-2164-13-S5-S3-S1.pdf]

# Stereotype Definitions for Biological Entity Classes

| Stereotype | Base Class | Description |
|---|---|---|
| Continuant<br><<continuant>> | Class | <<continuant>> represents a biological entity class. Each <<continuant>> instance represents a particular biological entity that continues to exist throughout time, independently of changes (including location). This entity exists in a given spatial region and at an instant of time. |
| **Notation** | **Examples** | |
| «continuant»<br>**A** | «continuant»<br>**Cell** | «continuant»<br>**DNA** |

| Stereotype | Base Class | Description |
|---|---|---|
| Material<br><<material>> | Continuant | <<material>> represents a specific type of <<continuant>>. Each <<material>> instance represents a continuant that has matter and exists in a given spatial region at an instant of time. |
| **Notation** | **Examples** | |
| «material»<br>**A** | «material»<br>**Cell** | «material»<br>**Hemoglobin** |

| Stereotype | Base Class | Description |
|---|---|---|
| Immaterial<br><<immaterial>> | Continuant | <<immaterial>> represents a specific type of <<continuant>>. Each <<immaterial>> instance represents a continuant that has no matter and exists as part of another continuant in a given spatial region at an instant of time. |
| **Notation** | **Examples** | |
| «immaterial»<br>**A** | «immaterial»<br>**Medullary Cavity** | «immaterial»<br>**Interior of Digestive Tube** |

| Stereotype | Base Class | Description |
|---|---|---|
| Process<br><<process>> | Class | <<process>> represents a biological entity class. Each <<process>> instance represents a particular biological process that unfolds into successive temporal phases. Namely, it has a beginning, middle and end, and occurs at an instant of time. |
| **Notation** | **Examples** | |
| «process»<br>**A** | «process»<br>**DNA Replication** | «process»<br>**Photosynthesis** |

# Stereotype Definitions for Foundational Relations

| Metaclass | Base Class | Description |
|---|---|---|
| *OBORelation* | *DirectedRelationship* | *OBO Relation* is an abstract metaclass that represents the properties of all binary and directed relations that apply to <<Continuant>>, <<Material>>, <<Immaterial>> and <<Process>> entity classes. |

| Metaclass | Base Class | Description |
|---|---|---|
| *FoundationalRelation* | *OBORelation* | *Foundational Relation* is an abstract metaclass that represents a specific type of *OBO Relation*. This metaclass represents the properties of the so-called foundational relations. |

| Stereotype | Base Class | Description |
|---|---|---|
| Instance_of<br><<instance_of>> | *FoundationalRelation*<br>Dependency | <<instance_of>> represents a relation between a biological entity class and an instance of this class. For continuants this relation is established between a class and an instance which it instantiates at a specific time. For processes this relation is established between a class and an instance which it instantiates holding independently of time. |

| Notation | Example |
|---|---|
| 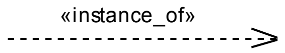 | 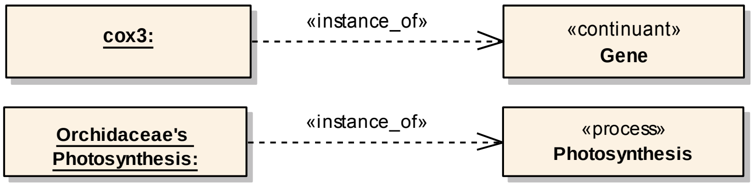 |

**Constraints**

[1] The two ends of dependency relations stereotyped as <<instance_of>> must have multiplicities equal to exactly 1:

context  Instance_of inv:
self.client->size() = 1 and self.supplier->size() = 1

[2] A dependency relation stereotyped as <<instance_of>> must have an instance specification on its source end and a class stereotyped as <<continuant>> or as <<process>> on its target end:

context Instance_of inv:
self.client->exists(x|x.oclIsKindOf(InstanceSpecification)) and
self.supplier->exists(x|x.oclIsKindOf(Continuant) or x.oclIsTypeOf(Process))

<table>
<tr><th>Stereotype</th><th>Base Classes</th><th>Description</th></tr>
<tr><td>Is_a<br><<is_a>></td><td>FoundationalRelation<br>Generalization</td><td><<is_a>> is a specific type of Foundational Relation that represents a relation between a source biological entity class that is a subtype of a target biological entity class, acting as a supertype. <<is_a>> expresses that any source instance also instantiates the target class. <<is_a>> can be interpreted as "is subtype of" and connects two continuants or two processes.</td></tr>
</table>

<table>
<tr><th>Notation</th><th>Examples</th></tr>
<tr><td>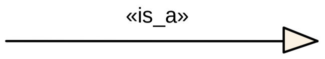
</td><td>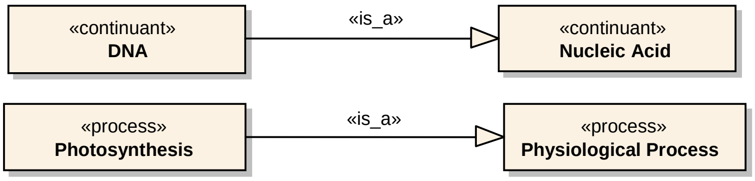
</td></tr>
</table>

**Constraints**

[1] A generalization relation stereotyped as <<is_a>> must have classes stereotyped as <<continuant>> or as <<process>> at both ends:

```
context Is_a inv:
  (self.general.oclIsKindOf(Continuant) and self.specific.oclIsKindOf(Continuant)) or
  (self.general.oclIsTypeOf(Process) and self.specific.oclIsTypeOf(Process))
```

[2] If a generalization relation stereotyped as <<is_a>> has a class stereotyped as <<material>> on its source or target end, its other end must have a class stereotyped as <<material>>:

```
context Is_a inv:
   if self.general.oclIsTypeOf(Material) then self.specific.oclIsTypeOf(Material) endif
   if self.specific.oclIsTypeOf(Material) then self.general.oclIsTypeOf(Material) endif
```

[3] If a generalization relation stereotyped as <<is_a>> has a class stereotyped as <<immaterial>> on its source or target end, its other end must have a class stereotyped as <<immaterial>>:

```
context Is_a inv:
   if self.general.oclIsTypeOf(Immaterial) then self.specific.oclIsTypeOf(Immaterial) endif
   if self.specific.oclIsTypeOf(Immaterial) then self.general.oclIsTypeOf(Immaterial) endif
```

| Stereotype | Base Classes | Description |
|---|---|---|
| Part_of<br><<part_of>> | *FoundationalRelation*<br>Association | <<part_of>> is a specific type of *Foundational Relation* that represents a relation between a source and a target biological entity class. Each instance of the source class is part of a “whole” represented by an instance of the target class. <<part_of>> connects two continuants or two processes. |

| Notation | Examples |
|---|---|
| 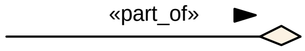 | 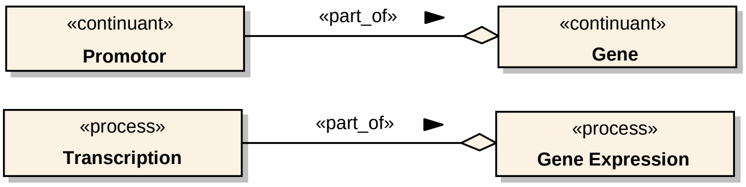 |

**Constraints**

[1] An association stereotyped as <<part_of>> has only two ends and one must be a shared aggregation:

```
context Part_of inv:
  self.memberEnd ->size() = 2 and self.memberEnd ->exists(x,y| x<>y and x.aggregation= #shared and y.aggregation= #none)
```

[2] Each instance of the source end of an association stereotyped as <<part_of>> must be associated to at least one instance of the target end:

```
context Part_of inv:
  self.source->forAll(x|x.part_of->exists(y|not y.target->isEmpty()))
```

[3] An association stereotyped as <<part_of>> must have classes stereotyped as <<continuant>> or as <<process>> at both ends:

```
context Part_of inv:
  self.source->exists(x|x.oclIsKindOf(Continuant)) and self.target->exists(y|y.oclIsKindOf(Continuant)) or
  self.source->exists(x|x.oclIsTypeOf(Process)) and self.target->exists(y|y.oclIsTypeOf(Process))
```

[4] If an association stereotyped as <<part_of>> has a class stereotyped as <<immaterial>> on its aggregate end, its other end must have a class stereotyped as <<immaterial>>:

```
context Part_of inv:
  if self.memberEnd->exists(x|x.aggregation=#shared and x.endType->exists(y|y.oclIsTypeOf(Immaterial)))
  then self.memberEnd->exists(x|x.aggregation=#none and x.endType->exists (y|y.oclIsTypeOf(Immaterial)))
  endif
```

[5] If an association stereotyped as <<part_of>> has a class stereotyped as <<material>> on its non-aggregate end, its other end must have a class stereotyped as <<material>>:

```
context Part_of inv:
  if self.memberEnd->exists(x|x.aggregation=#none and x.endType->exists (y|y.oclIsTypeOf(Material)))
  then self.memberEnd->exists(x|x.aggregation=#shared and x.endType->exists(y|y.oclIsTypeOf(Material)))
  endif
```

| Stereotype | Base Classes | Description |
|---|---|---|
| Has_part<br><<has_part>> | *FoundationalRelation*<br>Association | <<has_part>> is a specific type of *Foundational Relation* that represents the inverse of the relation <<part_of>>. |

| Notation | Examples |
|---|---|
| 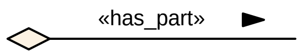  | 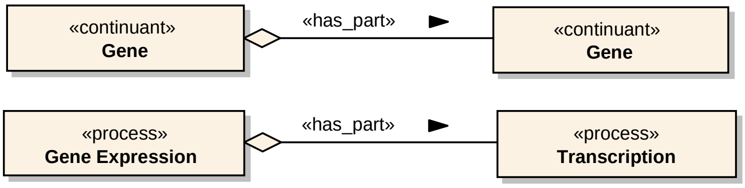  |

**Constraints**

[1] An association stereotyped as <<has_part>> has only two ends and one of them must be a shared aggregation:

```
context Has_part inv:
  self.memberEnd->size() = 2 and self.memberEnd->exists(x,y| x<>y and x.aggregation= #shared and y.aggregation= #none)
```

[2] Each instance of the source end of an association stereotyped as <<has_part>> must be associated to at least one instance of the target end:

```
context Has_part inv:
    self.source->forAll(x|x.has_part->exists(y|not y.target->isEmpty()))
```

[3] An association stereotyped as <<has_part>> must have classes stereotyped as <<continuant>> or as <<process>> at both ends:

```
context Has_part inv:
   self.source->exists(x| x.oclIsKindOf(Continuant)) and self.target->exists(y|y.oclIsKindOf(Continuant)) or
   self.source->exists(x| x.oclIsTypeOf(Process)) and self.target->exists(y|y.oclIsTypeOf(Process))
```

[4] If an association stereotyped as <<has_part>> has a class stereotyped as <<immaterial>> on its aggregate end, its other end must have a class stereotyped as <<immaterial>>:

```
context Has_part inv:
   if self.memberEnd->exists(x|x.aggregation=#shared and x.endType->exists(y|y.oclIsTypeOf(Immaterial)))
   then self.memberEnd->exists(x|x.aggregation=#none and x.endType->exists (y|y.oclIsTypeOf(Immaterial)))
   endif
```

[5] If an association stereotyped as <<has_part>> has a class stereotyped as <<material>> on its non-aggregate end, its other end must have a class stereotyped as <<material>>:

```
context Has_part inv:
   if self.memberEnd->exists(x|x.aggregation=#none and x.endType->exists (y|y.oclIsTypeOf(Material)))
   then self.memberEnd->exists(x|x.aggregation=#shared and x.endType->exists(y|y.oclIsTypeOf(Material)))
   endif
```

| Stereotype | Base Class | Description |
|---|---|---|
| Integral_part_of<br><<integral_part_of>> | Part_of | <<integral_part_of>> represents a specific type of <<part_of>> in which a source biological entity class is part of a target biological entity class (<<part_of>> relation) and at the same time the target class has the source class as its part (<<has_part>> relation). |

**Notation**

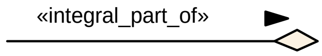


**Examples**

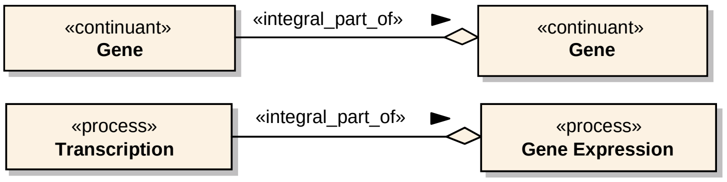


**Constraint**

[1] Each instance of the target end of an association stereotyped as <<integral_part_of>> must be associated to each instance of the source end through an association stereotyped as <<has_part>>:

```
context Integral_part_of inv:
  self.target->forAll(x|x.has_part->exists(y|y.target->exists(z|z.integral_part_of->exists(w|w.target->includes(x)))))
```

| Stereotype | Base Class | Description |
|---|---|---|
| Has_integral_part<br><<has_integral_part>> | Has_part | <<has_integral_part>> is a specific type of <<has_part>> that represents the inverse of the relation <<integral_part_of>>. |

**Notation**

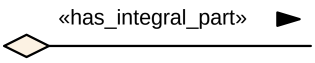


**Examples**

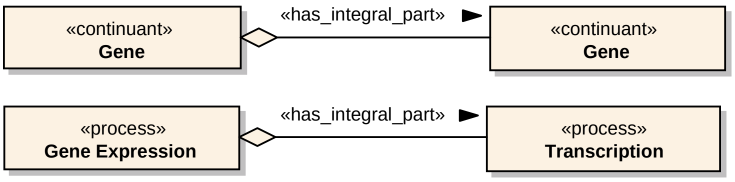


**Constraint**

[1] Each instance of the target end of an association stereotyped as <<has_integral_part>> must be associated to each instance of the source end through an association stereotyped as <<part_of>>:

```
context Has_integral_part inv:
  self.target->forAll(x|x.part_of->exists(y|y.target->exists(z|z.has_integral_part->exists(w|w.target->includes(x)))))
```

| Stereotype | Base Class | Description |
| --- | --- | --- |
| Proper_part_of<br><<proper_part_of>> | Part_of | <<proper_part_of>> is a specific type of <<part_of>> that associates a source biological entity class to a target biological entity class. Each instance of the source class is part of a “whole” represented by an instance of the target class. However, this relation has the additional constraint that the source and target classes must be distinct. |

| Notation | Examples |
| --- | --- |
| 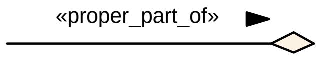 | 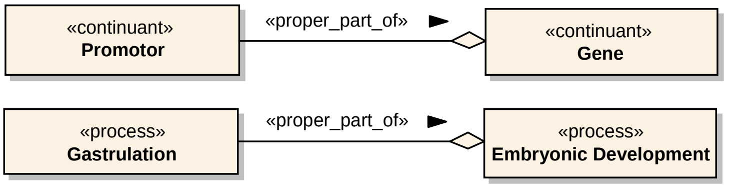 |

**Constraint**

[1] An association stereotyped as <<proper_part_of>> must have distinct classes in its source and target ends:

context Proper_part_of inv:
  self.source->exists(x|x->intersection(self.target)->isEmpty())

| Stereotype | Base Class | Description |
| --- | --- | --- |
| Has_proper_part<br><<has_proper_part>> | Has_part | <<has_proper_part>> is a specific type of <<has_part>> that represents the inverse of the relation <<proper_part_of>>. |

| Notation | Examples |
| --- | --- |
| 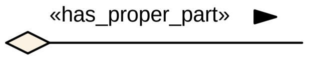 | 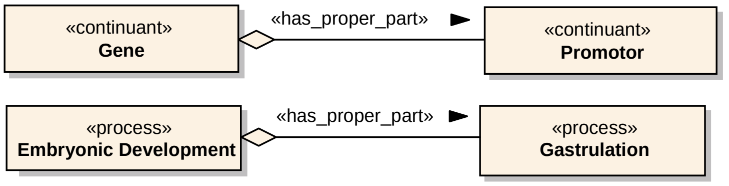 |

**Constraint**

[1] An association stereotyped as <<has_proper_part>> must have distinct classes in its source and target ends:

context Has_proper_part inv:
  self.source->exists(x|x->intersection(self.target)->isEmpty())

# Stereotype Definitions for Spatial Relations

| **Metaclass** | **Base Classes** | **Description** |
|---|---|---|
| *SpatialRelation* | *OBORelation*<br>Association | A *Spatial Relation* is an abstract metaclass that represents a specific type of *OBO Relation*. This metaclass represents the properties of all spatial relations that connect biological entity classes in terms of spatial regions occupied by their instances. |

**Constraints**

[1] Associations stereotyped by subtypes of *SpatialRelation* have two ends and only one of them is navigable:

```
context SpatialRelation inv:
  self.memberEnd->size() = 2 and self.navigableOwnedEnd->size() = 1
```

[2] Each instance of the source end of associations stereotyped by subtypes of *SpatialRelation* must be associated to at least one instance of the target end:

```
context SpatialRelation inv:
  self.source->forAll(x|x.spatialRelation->exists(y|not y.target->isEmpty()))
```

| **Stereotype** | **Base Class** | **Description** |
|---|---|---|
| Adjacent_to<br><<adjacent_to>> | *SpatialRelation* | <<adjacent_to>> represents a specific type of *Spatial Relation* that connects a source continuant to a target continuant. <<adjacent_to>> expresses that the spatial region occupied by a source continuant is adjacent to the spatial region occupied by a target continuant. |

**Notation**

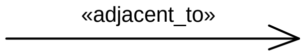


**Example**

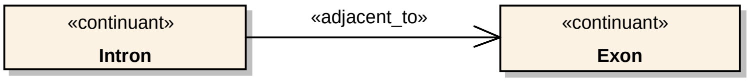


**Constraint**

[1] An association stereotyped as <<adjacent_to>> must have classes stereotyped as <<continuant>> at both ends:

```
context Adjacent_to inv:
  self.source->exists(x|x.oclIsKindOf(Continuant)) and self.target->exists(y|y.oclIsKindOf(Continuant))
```

| **Stereotype** | **Base Class** | **Description** |
|---|---|---|
| Located_in<br><<located_in>> | *SpatialRelation* | <<located_in>> represents a specific type of *Spatial Relation* that connects two continuants (source and target). <<located_in>> expresses that at a given instant of time, a source continuant is located in a target continuant since the region occupied by the source instance is part of (is in) the region occupied by the target instance at this instant of time. |

**Notation**

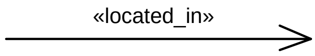


**Example**

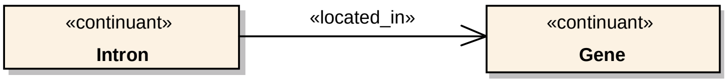


**Constraint**

[1] An association stereotyped as <<located_in>> must have classes stereotyped as <<continuant>> at both ends:

```
context Located_in inv:
  self.source->exists(x|x.oclIsKindOf(Continuant)) and self.target->exists(y|y.oclIsKindOf(Continuant))
```

| Stereotype | Base Class | Description |
|---|---|---|
| Location_of<br><<location_of>> | *SpatialRelation* | <<location_of>> is a specific type of *Spatial Relation* that represents the inverse of the relation <<located_in>>. |

| Notation | Example |
|---|---|
| 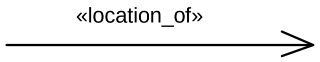 | 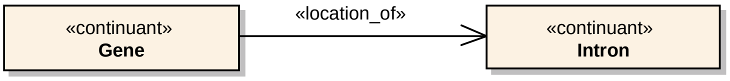 |

**Constraint**

[1] An association stereotyped as <<location_of>> must have classes stereotyped as <<continuant>> at both ends:

context Location_of inv:
self.source->exists(x|x.oclIsKindOf(Continuant)) and self.target->exists(y|y.oclIsKindOf(Continuant))

| Stereotype | Base Class | Description |
|---|---|---|
| Contained_in<br><<contained_in>> | *SpatialRelation* | <<contained_in>> represents a specific type of *Spatial Relation* that connects a source material continuant to a target immaterial continuant. <<contained_in>> expresses that a source continuant is contained in a target continuant, but the source instance is not part of the target instance. |

| Notation | Example |
|---|---|
| 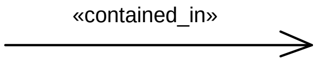 | 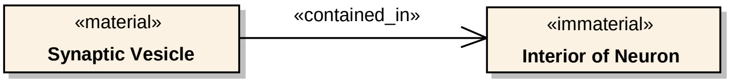 |

**Constraint**

[1] An association stereotyped as <<contained_in>> must have a class stereotyped as <<material>> on its non-navigable end and a class stereotyped as <<immaterial>> on its navigable end:

context Contained_in inv:
(self.memberEnd - self.navigableOwnedEnd)->exists(x|x.endType->exists(y|y.oclIsTypeOf(Material))) and
self.navigableOwnedEnd->exists(x|x.endType->exists(y|y.oclIsTypeOf(Immaterial)))

| Stereotype | Base Class | Description |
|---|---|---|
| Contains<br><<contains>> | *SpatialRelation* | <<contains>> is a specific type of *Spatial Relation* that represents the inverse of the relation <<contained_in>>. |

| Notation | Example |
|---|---|
| 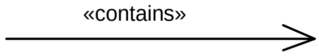 | 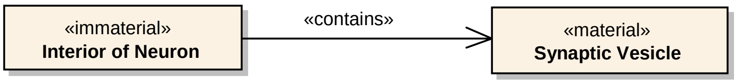 |

**Constraint**

[1] An association stereotyped as <<contains>> must have a class stereotyped as <<immaterial>> on its non-navigable end and a class stereotyped as <<material>> on its navigable end:

context Contains inv:
(self.memberEnd - self.navigableOwnedEnd)->exists(x|x.endType->exists(y|y.oclIsTypeOf(Immaterial))) and
self.navigableOwnedEnd->exists(x|x.endType->exists(y|y.oclIsTypeOf(Material)))

# Stereotype Definitions for Temporal Relations

| Metaclass | Base Classes | Description |
|---|---|---|
| *TemporalRelation* | *OBORelation*<br>Association | *Temporal Relation* is an abstract metaclass that represents a specific type of *OBO Relation*. This metaclass represents the properties of all temporal relations that connect biological entity classes whose instances exist at different instants of time. |

**Constraints**

[1] Associations stereotyped by subtypes of *TemporalRelation* have two ends and only one of them is navigable:

```
context TemporalRelation inv:
  self.memberEnd ->size() = 2 and self.navigableOwnedEnd->size()=1
```

[2] Each instance of the source end of associations stereotyped by subtypes of *TemporalRelation* must be associated to at least one instance of the target end:

```
context TemporalRelation inv:
  self.source->forAll(x|x.temporalRelation->exists(y|not y.target->isEmpty()))
```

| Stereotype | Base Class | Description |
|---|---|---|
| Transformation_of<br><<transformation_of>> | *TemporalRelation* | <<transformation_of>> represents a specific type of *Temporal Relation* that connects a source material continuant to a target material continuant. <<transformation_of>> expresses that some material continuant instantiates different entity classes at different instants of time. |

| Notation | Example |
|---|---|
| 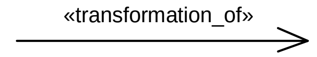  | 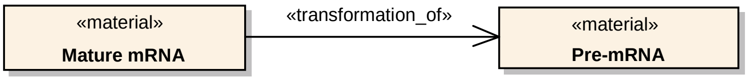  |

**Constraint**

[1] An association stereotyped as <<transformation_of>> must have classes stereotyped as <<material>> at both ends:

```
context Transformation_of inv:
  self.source->exists(x|x.oclIsTypeOf(Material)) and self.target->exists(y|y.oclIsTypeOf(Material))
```

| Stereotype | Base Class | Description |
|---|---|---|
| Derives_from<br><<derives_from>> | *TemporalRelation* | <<derives_from>> represents a specific type of *Temporal Relation* that connects a source material continuant to a target material continuant. <<derives_from>> expresses that a source continuant immediately derives from a target continuant. Thus, the source continuant begins to exist at the same instant of time the target continuant ceases to exist and a portion of the target matter is inherited by the source. |

| Notation | Example |
|---|---|
| 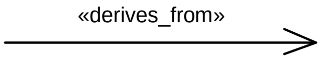  | 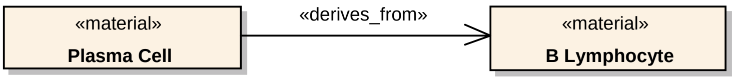  |

**Constraint**

[1] An association stereotyped as <<derives_from>> must have classes stereotyped as <<material>> at both ends:

```
context Derives_from inv:
  self.source->exists(x|x.oclIsTypeOf(Material)) and self.target->exists(y|y.oclIsTypeOf(Material))
```

| Stereotype | Base Class | Description |
|---|---|---|
| Derived_into<br><<derived_into>> | *TemporalRelation* | <<derived_into>> is a specific type of *Temporal Relation* that represents the inverse of the relation <<derives_from>>. |
| **Notation** | **Example** | |
| 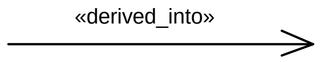 | 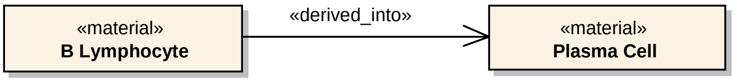 | |
| **Constraint** | | |
| [1] An association stereotyped as <<derived_into>> must have classes stereotyped as <<material>> at both ends:<br><br>context Derived_into inv:<br>self.source->exists(x\|x.oclIsTypeOf(Material)) and self.target->exists(y\|y.oclIsTypeOf(Material)) | | |

| Stereotype | Base Class | Description |
|---|---|---|
| Preceded_by<br><<preceded_by>> | *TemporalRelation* | <<preceded_by>> represents a specific type of *Temporal Relation* that connects a source process to a target process. <<preceded_by>> expresses that a source process occurs in an instant of time prior to the occurrence of a target process. |
| **Notation** | **Example** | |
| 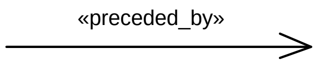 | 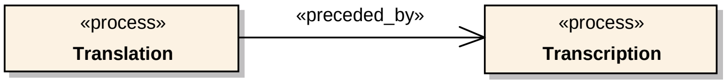 | |
| **Constraint** | | |
| [1] An association stereotyped as <<preceded_by>> must have classes stereotyped as <<process>> at both ends:<br><br>context Preceded_by inv:<br>self.source->exists(x\|x.oclIsTypeOf(Process)) and self.target->exists(y\|y.oclIsTypeOf(Process)) | | |

| Stereotype | Base Class | Description |
|---|---|---|
| Precedes<br><<precedes>> | *TemporalRelation* | <<precedes>> is a specific type of *Temporal Relation* that represents the inverse of the relation <<preceded_by>>. |
| **Notation** | **Example** | |
| 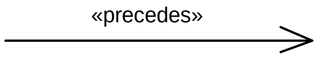 | 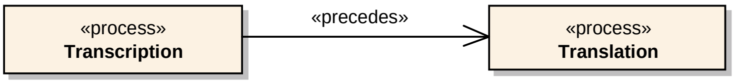 | |
| **Constraint** | | |
| [1] An association stereotyped as <<precedes>> must have classes stereotyped as <<process>> at both ends:<br><br>context Precedes inv:<br>self.source->exists(x\|x.oclIsTypeOf(Process)) and self.target->exists(y\|y.oclIsTypeOf(Process)) | | |

# Stereotype Definitions for Participation Relations

| Metaclass | Base Classes | Description |
|---|---|---|
| *ParticipationRelation* | *OBORelation*<br>Association | *Participation Relation* is an abstract metaclass that represents a specific type of *OBO Relation*. This metaclass represents the properties of all participation relations of continuants in processes. |

**Constraints**

[1] Associations stereotyped by subtypes of *ParticipationRelation* have two ends and only one of them is navigable:

```
context ParticipationRelation inv:
  self.memberEnd ->size() = 2 and self.navigableOwnedEnd->size()=1
```

[2] Each instance of the source end of associations stereotyped by subtypes of *ParticipationRelation* must be associated to at least one instance of the target end:

```
context ParticipationRelation inv:
  self.source->forAll(x|x.participationRelation->exists(y|not y.target->isEmpty()))
```

| Stereotype | Base Class | Description |
|---|---|---|
| Has_participant<br><<has_participant>> | *ParticipationRelation* | <<has_participant>> represents a specific type of *Participation Relation* that connects a source process to a target continuant. <<has_participant>> expresses that a source process has a target continuant as its participant at any instant of time. |

**Notation**

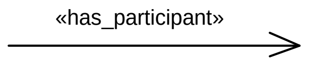


**Example**

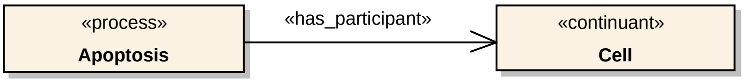


**Constraint**

[1] An association stereotyped as <<has_participant>> must have a class stereotyped as <<continuant>> on its navigable end and must have a class stereotyped as <<process>> on its non-navigable end:

```
context Has_participant inv:
  self.navigableOwnedEnd->exists(x|x.endType->exists(y|y.oclIsKindOf(Continuant))) and
   (self.memberEnd - self.navigableOwnedEnd)->exists(x|x.endType->exists(y|y.oclIsTypeOf(Process)))
```

| Stereotype | Base Class | Description |
|---|---|---|
| Participates_in<br><<participates_in>> | *ParticipationRelation* | <<participates_in>> is a specific type of *Participation Relation* that represents the inverse of the relation <<has_participant>>. |

**Notation**

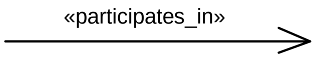


**Example**

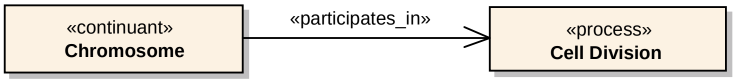


**Constraint**

[1] An association stereotyped as <<participates_in>> must have a class stereotyped as <<process>> on its navigable end and must have a class stereotyped as <<continuant>> on its non-navigable end:

```
context Participates_in inv:
  self.navigableOwnedEnd->exists(x|x.endType->exists(y|y.oclIsTypeOf(Process))) and
  (self.memberEnd - self.navigableOwnedEnd)->exists(x| x.endType->exists(y|y.oclIsKindOf(Continuant)))
```

| Stereotype | Base Class | Description |
| --- | --- | --- |
| Has_agent<br><<has_agent>> | Has_participant | <<has_agent>> represents a specific type of relation <<has_participant>>. <<has_agent>> connects a source process to a target material continuant. A target continuant is responsible for the occurrence (execution) of a source process. |

| Notation | Example |
| --- | --- |
| 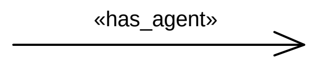 | 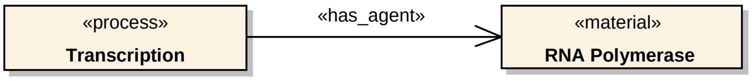 |





**Constraint**

[1] An association stereotyped as <<has_agent>> must have a class stereotyped as <<material>> on its navigable end:

```
context Has_agent inv:
  self.navigableOwnedEnd->exists(x|x.endType->exists(y|y.oclIsTypeOf(Material)))
```

| Stereotype | Base Class | Description |
| --- | --- | --- |
| Agent_in<br><<agent_in>> | Participates_in | <<agent_in>> is a specific type of <<participates_in>> that represents the inverse of the relation <<has_agent>>. |

| Notation | Example |
| --- | --- |
| 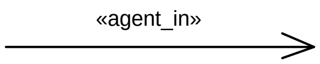 | 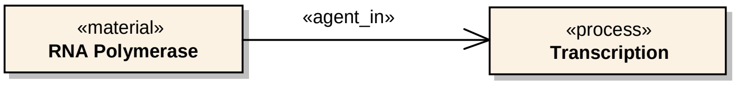 |





**Constraint**

[1] An association stereotyped as <<agent_in>> must have a class stereotyped as <<material>> on its non-navigable end:

```
context Agent_in inv:
  (self.memberEnd - self.navigableOwnedEnd)->exists(x|x.endType->exists(y|y.oclIsTypeOf(Material)))
```
